# The UK Medical Research Council and clinical trials, 1934-1960

**DOI:** 10.1186/1745-6215-14-S1-I1

**Published:** 2013-11-29

**Authors:** Iain Chalmers

**Affiliations:** 1 James Lind Initiative, Oxford, UK

## 

The UK Medical Research Council (MRC) is 100 years old this year. Its ‘Centenary Timeline' contains the following brief reference to the Council's role in the development of clinical trials:

## 1940-1949: Randomised controlled trial design pioneered

MRC scientists developed what is today the gold standard for clinical trial design while testing streptomycin to treat pulmonary tuberculosis.

Although the MRC failed to make any substantive contribution to the development of trial methods before the 1940s, it made exceptionally important contributions between 1944 and 1960. Surprisingly, the Council has been curiously silent about the enduring value of its role in developing clinical trial methods, and about the important methodological legacy left by the director of its Statistical Research Unit - Austin Bradford Hill. Stephen Lock, a former editor of the *British Medical Journal*, has suggested that ‘the randomised controlled trial is a British invention' and that Bradford Hill should have been awarded a Nobel prize for his key role in helping to put medicine on a rational scientific footing. Some of us think that it is high time that the Council made more of its historic achievement.

For details see: Chalmers I (2013). The MRC and multicentre clinical trials: from a damning report to international recognition. JLL Bulletin: Commentaries on the history of treatment evaluation (http://www.jameslindlibrary.org).

